# Long Noncoding RNA and Epithelial Mesenchymal Transition in Cancer

**DOI:** 10.3390/ijms20081924

**Published:** 2019-04-18

**Authors:** Mila Gugnoni, Alessia Ciarrocchi

**Affiliations:** Laboratory of Translational Research, Azienda Unità Sanitaria Locale-IRCCS di Reggio Emilia, 42122 Reggio Emilia, Italy; Alessia.Ciarrocchi@ausl.re.it

**Keywords:** long noncoding RNAs (lncRNAs), Epithelial to Mesenchymal Transition (EMT), cancer

## Abstract

Epithelial–mesenchymal transition (EMT) is a multistep process that allows epithelial cells to acquire mesenchymal properties. Fundamental in the early stages of embryonic development, this process is aberrantly activated in aggressive cancerous cells to gain motility and invasion capacity, thus promoting metastatic phenotypes. For this reason, EMT is a central topic in cancer research and its regulation by a plethora of mechanisms has been reported. Recently, genomic sequencing and functional genomic studies deepened our knowledge on the fundamental regulatory role of noncoding DNA. A large part of the genome is transcribed in an impressive number of noncoding RNAs. Among these, long noncoding RNAs (lncRNAs) have been reported to control several biological processes affecting gene expression at multiple levels from transcription to protein localization and stability. Up to now, more than 8000 lncRNAs were discovered as selectively expressed in cancer cells. Their elevated number and high expression specificity candidate these molecules as a valuable source of biomarkers and potential therapeutic targets. Rising evidence currently highlights a relevant function of lncRNAs on EMT regulation defining a new layer of involvement of these molecules in cancer biology. In this review we aim to summarize the findings on the role of lncRNAs on EMT regulation and to discuss their prospective potential value as biomarkers and therapeutic targets in cancer.

## 1. Epithelial to Mesenchymal Transition

The epithelial to mesenchymal transition (EMT) is a multistep, plastic and reversible process that allows epithelial cells to acquire mesenchymal characteristics. Downregulation of cell-adhesion molecules like epithelial cadherins, occludins, claudins and cytokeratins, together with the coordinated upregulation of mesenchymal cadherins, vimentin and matrix metalloproteinases (MMPs), promote loss of cell–cell adhesion and apico-basal polarity and acquisition of invasive and migratory capacity [1,2,3]. The trans-differentiation of epithelial cells is induced by many pleiotropic signals including growth factors (transforming growth factor beta TGFβ, epidermal growth factor EGF, vascular endothelial growth factor VEGF, fibroblast growth factor FGF). Among them, TGFβ is the major player orchestrating EMT, through SMAD-dependent (canonical) or independent (non-canonical) pathways. Activation of several intracellular signaling pathways (including mitogen-activated protein kinase MAPK, phosphoinositide 3-kinases PI3K, Hedgehog, Notch and wingless/integrated Wnt) promotes the expression of specific transcription factors (EMT-TFs), noncoding RNAs (ncRNAs), epigenetic and post-translational modificators that together orchestrate a deep gene expression reprogramming. EMT-TFs are a group of transcription factors among which SNAIL, SLUG, TWIST, zinc finger E-box-binding homeobox 1 and 2 (ZEB1, ZEB2) are well known to cooperate to a different extent in distinct cellular contexts to inhibit or induce the expression of a precise set of genes indispensable for EMT partial or complete execution.

EMT is fundamental during embryogenesis but, aberrantly reactivated, it plays a relevant role basically at any stage of cancer progression, from tumorigenesis to growth, invasion, metastasization and resistance to therapy [1,2,3]. EMT is a transitory state and during cancer metastatic colonization, its reverse, the mesenchymal to epithelial transition (MET) is equally fundamental to complete colonization of distant sites, blocking migration to sustain proliferation [4]. Erroneously, EMT and MET are often considered as stand-alone and mutually exclusive phenotypes. Instead, full EMT is rarely achieved and during this transition cells reside in a plethora of intermediate states that contribute to cancer heterogeneity [5].

This plasticity is granted by the capacity of cancer cells to modulate gene expression, transitioning from different states of partial EMT. For example, in well differentiated carcinomas, cells at the invasion front often migrate in groups instead of as single cell, a phenomenon called collective cell migration [6,7,8]. In this state, cells acquire altered apico-basal polarity, extracellular matrix and invasion and motility capacity, but remain connected to each other by epithelial Cadherins and cell-cell junctions [7,8]. EMT induction in cancer depends on complex, tissue-specific networks. Understanding all the aspects regulating this process could provide useful tools to counteract it in tumor cells, but also to gain molecular information to distinguish aggressive cells prone to form metastasis.

## 2. Noncoding RNAs Outbreak

The great effort made to understand organization and function of the human genome led to the surprising discovery that 70–90% of the DNA is transcribed but only 2% encodes for proteins [9,10,11].

Recently, thanks to improved sequencing technologies, we are reinterpreting our knowledge about noncoding DNA and its function, discovering that a large part of it is transcribed in noncoding RNAs (ncRNAs) [12,13].

It is indeed clear that the human transcriptome consists of several types of ncRNAs which cover a plethora of functions. ncRNAs are commonly divided based on their length in long-noncoding RNAs (lncRNAs) which are longer than 200 bp, and small-ncRNAs, among which small inhibitory RNAs (siRNAs), small nuclear RNAs (snRNAs), small nucleolar RNAs (snoRNAs), piwi-interacting RNAs (piRNAs), which are shorter than 200 bp and play a variety of cellular functions [13].

## 3. lncRNAs

lncRNAs are longer than 200 bp and share common features with mRNAs, like exon-intron structure, transcription by RNA Polymerase II, post-transcriptional processing (5′ cap, polyadenylation and splicing), similar organization of the 3′UTR regions and secondary structures, sequence composition and thermodynamic parameters. On the contrary, lncRNAs conservation between species and their expression are usually lower compared to mRNAs. The number of lncRNAs is predicted to be from 3 to 100-fold higher than the number of mRNAs, and their localization can be nuclear, chromatin associated, but also cytoplasmic [14]. Based on the position and direction of transcription relative to the nearest gene, lncRNAs are broadly divided in sense, antisense, bidirectional, intronic and long intergenic [14,15,16]. 

Since transcriptional regulatory elements such enhancers and promoters can be transcribed bi-directionally, it is possible that many lncRNAs are side products of the act of transcription and do not have specific functions [17,18,19]. It has also been reported that some transcripts annotated as lncRNAs encode for small proteins. However, stability and function of these small peptides is questioned [20,21]. 

Thus, rigorous functional annotation of these molecules is required to fully understand whether and in which extent lncRNAs participate to cellular functions.

The biological relevance of lncRNAs must be experimentally determined case by case. Improvement of technical skills will be essential to study these molecules, since appropriate functional assays are still limited. Nevertheless, an increasing number of works underline how lncRNAs can have central roles in a great variety of both physiological and pathological processes, as cell differentiation, tumorigenesis, metastasization, immune response, aging and others [22,23,24,25,26,27,28].

**Figure 1:**
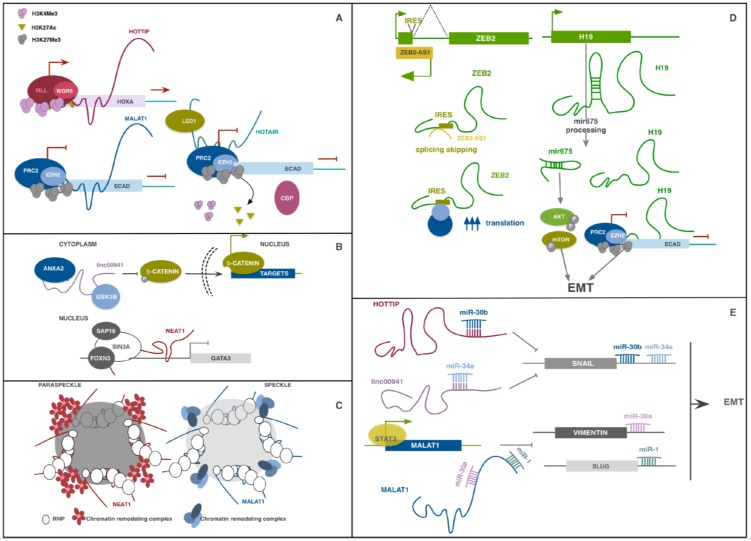
Schematic representation of different molecular mechanisms through which lncRNAs regulate EMT in cancer. (**A**) *Examples of lncRNAs in epigenetic regulation*. HOTTIP binds WDR5, part of the MLL complex, on the HOXA genes locus to promote H3K4me3 modification enhancing transcription. MALAT1 interacts with EZH2, part of the inhibiting complex PRC2, on E-CAD promoter. Subsequent tri-methylation of H3K27 impedes E-CAD transcription. HOTAIR can bind both LSD1 and PRC2 complexes on E-CAD promoter to transcriptionally silence it. Their binding provokes the displacement of CBP acetyl-transferase, switching H3K27 acetylation to methylation, and the de-methylation of H3K4. (**B**) *Examples of lncRNAs in protein scaffolding*. In the cytoplasm, linc00941 helps ANXA2 and GSKβ interaction which leads to β-Catenin phosphorylation inhibition, promoting its nuclear localization and the expression of its target genes. In a similar way, in the nucleus, NEAT1 functions as a scaffold for FOXN3 and SIN3A to inhibit GATA3 transcription. (**C**) *Examples of lncRNAs in nuclear structures*. NEAT1 and MALAT1 participate to maintain nuclear sub-structures, paraspeckles and speckles respectively, also contributing to their function. (**D**) *Examples of lncRNAs and transcript processing*. ZEB2-AS1 is an antisense lncRNA overlapping the 5’UTR of the ZEB2 gene. Its sequence is complementary to a splicing site of an intron containing an IRES region. ZEB2-AS1-mediated splicing skipping preserves the IRES element, directly enhancing ZEB2 translation. H19 lncRNAs is itself a reservoir of another ncRNA, miR-675, which, once processed, potentiate its pro-EMT role. Indeed, while H19 epigenetically impede E-CAD transcription, miR-675 positively regulates AKT/mTOR axis. (**E**) *Examples of lncRNAs as ceRNAs*. HOTTIP and linc00941 participate to SNAIL upregulation sponging miR-30b and miR-34a, respectively. MALAT1, whose transcription is enhanced by STAT3, promotes EMT by sponging miR-30a and miR-1, thus upregulating VIMENTIN and SLUG levels.

lncRNAs have been reported to partake to gene regulation both in cis and in trans. Mature lncRNA transcripts can regulate the expression of nearby genes in cis or genes located far from their locus of origin in a sequence-specific manner, regulating chromatin structure and organization, directly binding promoters or enhancer regions, or facilitating the formation of complexes which support or inhibit active transcription [14,15]. Hundreds of lncRNAs are now described as mediators of epigenetic silencing through the recruitment of complexes like Polycomb Repressive Complex 2 (PRC2). This complex is responsible for the trimethylation of lysine 27 on histone 3 (H3K27me3), a repressive modification which silence the transcription of the genic locus (Figure 1A) [28,29]. lncRNAs can also participate to RNA splicing, editing, localization, stabilization, translation and degradation (Figure 1D). Some lncRNAs help to position specific chromatin regions in subcellular structures as speckles and paraspeckles to facilitate the action of transcription and splicing factors (Figure 1C) [30,31,32]. Another common mechanism of gene regulation by lncRNAs is their action as competing endogenous RNAs (ceRNAs). ceRNAs are molecules able to bind principally complementary miRNA response elements (MREs) impeding the binding of miRNAs on interacting molecules in a stoichiometric manner. miRNAs bind on the 3′UTR of mRNAs inhibiting their translation. Many miRNA classes are described as oncosuppressors for exerting their inhibitory function on EMT-inducing transcription factors. Their action is counteracted by EMT-promoting lncRNAs which, functioning as ceRNAs, sequester miRNAs, inhibiting their function (Figure 1E) [33,34,35].

Finally, lncRNAs can directly bind RNA-binding proteins (RBPs) and act like a guide to direct them to specific DNA loci or like a scaffold, to help the assembly of large complexes [26,27,28].

Given the variety of processes in which lncRNAs are involved, one of the most intriguing challenge nowadays is to understand their role as potential new biomarkers and drug targets.

## 4. lncRNAs and EMT

An increasing number of studies support the role of lncRNAs in the regulation of tumor progression and metastasization through the regulation of EMT. Although lncRNAs are usually divided in two categories in this field, the EMT promoters (pro-EMT) and the EMT-suppressors (anti-EMT), some of them have controversial functions in different type of tumors or different conditions, once more underlying the complexity and plasticity of tumor cells (Table 1).

## 5. Pro-EMT lncRNAs

TGFβ is one of the principal growth factors inducing a signaling cascade leading to EMT initiation. Its binding to TGFβ type I and type II serine-threonine kinase receptors provokes a change in the gene expression, inducing EMT-TFs but also a long list of lncRNAs. An increasing number of lncRNAs are described as regulators of TGFβ/SMAD signaling [36], among these, the Plasmacytoma variant translocation1 (**PVT1**). This lncRNA is transcribed from a genomic region frequently amplified in tumors that also comprise the c-MYC oncogene genomic locus. Its expression associates with metastasis and advanced stage and it acts favoring EMT and cell invasion and migration through several mechanisms, among which positively affecting SMAD2/3 phosphorylation and activation [37]. Its overexpression was also reported to be associated to p21 downregulation, a major effector in p53-mediated cell cycle modulation, promoting EMT in pancreatic cancer cells [38].

A recently described lncRNA involved in TGFβ-mediated EMT in non-small cell lung cancer (NSCLC) is the small nucleolar RNA host gene 1 lncRNA (**lnc-SNHG1**). Its expression is higher in tumor cell lines than in non-cancer ones and it was positively correlated with invasiveness. On the other side, its expression negatively correlated with miR-497, which is known to suppress cell proliferation, migration and invasion. Sponging miR-497, lnc-SNHG1 also regulates the expression of the insulin-like growth factor 1 receptor (IGF1-R) [39]. In parallel, another study on invasive pituitary tumors, reported an upregulation of this lncRNA in tumor cells. lnc-SNHG1 promoted cell proliferation acting on cell cycle and inhibiting apoptosis. Its effect on cell migration and invasion is executed by the upregulation of EMT markers and by the binding to miR-302/372/373/520, known inhibitors of SMAD3 and Wnt/β-catenin signaling pathways [40].

lncRNA-activated by TGFβ (**lncRNA-ATB**) was found upregulated in many tumor cell types among which thyroid, breast, gastric, hepatocellular cancer and plays a role in their initial tissue invasion and late colonization [41,42,43,44]. Some studies described that lncRNA-ATB acts as ceRNA for members of the miR-200 family members, which in turn are suppressors of ZEB1 and ZEB2 EMT-TFs, forming a positive feedback loop which further promotes the expression of TGFβ1 and TGFβ2 [44]. Moreover, lncRNA-ATB was found to bind Interleukin-11 (IL-11) mRNA, stabilizing it and promoting Signal transducer and activator of transcription 3 (STAT3) invading signaling [45]. Recent works suggest that lncRNA-ATB functions as ceRNA also for miR-141-3p, a direct binder and inhibitor of ZEB1 and ZEB2, in breast and gastric cancer [42,43] and that its expression correlates to trastuzumab resistance in HER2-positive breast cancer cells [46].

Several lncRNAs were described to interact miR-200 family, thus inhibiting their anti-EMT action, to induce ZEB1/2 upregulation in several types of cancer, one of these is the HOXA transcript induced by TGFβ (**lncRNA-HIT**) [47,48]. This lncRNA is transcribed from the homeobox (HOXA) family locus, and its expression was found particularly induced in a highly metastatic mammary cancer model. lncRNA-HIT depletion in these cells decreased cell migration, invasion, growth, and metastasis formation capacity and one of its major targets is E-cadherin (E-CAD). In vivo, lncRNA-HIT overexpression was more frequently detected in invasive than non-invasive breast carcinoma and associated with tumor progression [49].

Recently, lncRNA-HIT was also found in a complex with the cell cycle-controlling transcription factor E2F1, participating to the regulation of its target genes in a model of NSCLC, underlying that lncRNAs can have multiple and tissue-specific functions [50].

lncRNA-HIT is not the only lncRNA being transcribed from the HOXA family locus. The most studied are the antisense lncRNA **HOXA11-AS** and the near HOXA Distal Transcript Antisense RNA (**HOTTIP**). The first was originally described in cervical cancer as related to tumor progression, EMT induction and poor prognosis [51]. Its pro-invasive role was then confirmed in breast cancer, hepatocellular carcinoma, NSCLC, where it was found as ceRNA for several anti-EMT miRNA families [33,52,53]. HOTTIP was described by Wang and colleagues to modulate HOXA transcription in fibroblasts by targeting WDR5, a component of the mixed lineage leukemia (MLL) complex driving H3K4 trimethylation (Figure 1A) [54]. A lot of studies observed that its expression is induced in numerous cancer types, where it regulates several genes of the homeobox family. A study performed on esophageal squamous carcinoma (ESCC) cells demonstrated that HOTTIP functions as ceRNA for miR-30b thus regulating the repression of HOXA13 miR-30b-mediated, resulting in a positive HOTTIP/HOXA13 correlation. Its binding to this miRNA also upregulates SNAIL levels, promoting EMT and invasion properties (Figure 1E). HOTTIP direct binding to WDR5 and H3K4 trimethylation was further confirmed in this model for HOXA13 gene transcription regulation [55]. Recently, HOTTIP was also described to drive HOXA9 expression by the same WDR5-mediated mechanism and enhance the Wnt/β-catenin pathway in prostate cancer stem cells [56].

Among the great variable family of lncRNAs, some were discovered for their role in the structure and function of nuclear speckles and paraspeckles, discrete adjacent structures in the interchromatin nucleoplasmic space involved in RNA splicing processes [30]. Nuclear paraspeckle assembly transcript 1 (**NEAT1**) and metastasis-associated lung adenocarcinoma transcript 1 (**MALAT1**), also known as Nuclear-Enriched Abundant Transcript 2 (NEAT2) were among the first lncRNAs to be implicated in progression and metastasization of cancers [57,58,59,60,61]. Both these lncRNAs were originally characterized for their role in nuclear speckles and paraspeckles structure and function but were recently found to play a role in transcription regulation (Figure 1C) [31,32]. A recent work by Li et al. demonstrated that NEAT1 forms a complex with Forkhead Box N3 (FOXN3) and SIN3 Transcription Regulator Family Member A (SIN3A) which promotes EMT and invasion of breast cancer in vitro and in vivo (Figure 1B) [62]. Overexpression of both FOXN3 and NEAT1 in breast cancer led to GATA3 expression impairment and strongly correlated with poor prognosis.

MALAT1 expression has been linked to EMT promotion in several cell types [61,63] and its role on the process seemed contradictory, especially in breast cancer cells [64]. However, the great majority of works indicate that this lncRNA correlates with poor prognosis and cancer aggressiveness. Several works described its capacity to function as ceRNA for different classes of miRNAs involved in EMT regulation, as miR-205, which targets ZEB1 and ZEB2, miR-204 and miR-1, SLUG repressors (Figure 1E) [65,66,67,68]. Moreover, in a recent work, Wang et al. reported that TGFβ promotes overexpression of STAT3 which directly binds MALAT1 promoter to induce its transcription in head and neck squamous cell carcinoma (HNSCC). MALAT1, in turn, acts as ceRNA directly binding miR-30a, a well-known tumor suppressor miRNA, inhibiting its function [69].

Other studies described its role in modulating alternative splicing of EMT-related genes. In ovarian cancer, MALAT1 knockdown decreased proliferation, invasion, anchorage-independent growth, and decreased expression of RNA Binding Fox-1 Homolog 2 (RBFOX2), an RNA-binding protein which regulates alternative splicing events. RBFOX2 suppression resulted in preferential splicing of the pro-apoptotic isoform of KIF1B and increased anoikis [70]. Another mechanism by which MALAT1 partakes to EMT regulation is by recruiting chromatin-modifiers. In renal cancer, MALAT1 has been shown to bind to the Enhancer of zeste homolog 2 (EZH2), a component of the histone-lysine N-methyltransferase PRC2. Their binding on E-CAD promoter induces its methylation and repression, supporting the EMT process (Figure 1A) [29].

**ZEB1-AS1** and **ZEB2-AS1**, also known as ZEB2 natural antisense transcript (ZEB2NAT), are lncRNAs regulating the expression of ZEB1, ZEB2 and, indirectly, their target genes. ZEB1-AS1 can bind ZEB1 promoter region and recruit histone methyltransferase MLL1, activating its transcription. This lncRNA overexpression was also reported to cooperate to the induction of N-CAD and of metalloproteases MMP2 and MMP9, as well as to repression of E-CAD transcription and function. ZEB1-AS1 also functions as ceRNA for several oncosuppressive miRNAs, partaking to promote cell proliferation, migration and invasion [34,71,72] which correlates with increased tumor metastasization and shorter overall survival in several types of tumor [73,74]. The mechanism of action of ZEB2-AS1 is particularly interesting; its sequence is complementary to a splice site within an intron at the 5′ UTR of ZEB2 that contains an internal ribosome entry site (IRES). Binding of ZEB2-AS1 prevents splicing of this region, improving ZEB2 translation efficiency (Figure 1D). ZEB2-AS1 upregulation is partially responsible for ZEB2 overexpression and EMT promotion in several cancers [75,76].

Urothelial cancer associated 1 (**UCA1**) is another lncRNA shown to upregulate ZEB1 and ZEB2, promoting EMT. UCA1 is reported to sponge several miRNAs which control EMT-related JAG/Notch, Wnt- β Catenin, FGFR1/ERK pathways, in different tumors [77,78]. One of its targets is miR-145, which is targeted by several lncRNAs. Among these, Taurine upregulated gene1 (**TUG1**) lncRNA, which asserts pro-EMT functions in bladder cancer [79]. In a recent work, Wu et al. showed that UCA1 knock-down resulted in diminished proliferation, invasion, migration, and drug resistance. Interestingly, they found that also Autophagy-related 7 (ATG7) can be down-regulated by UCA1 knock-down or miR-582-5p, an UCA1 target, overexpression, leading to autophagy impairment, shedding light to the variety of pathways on which lncRNAs are implicated [80].

A well-studied lncRNA implicated in EMT in cancer is HOX transcript antisense RNA (**HOTAIR**). It exhibits cell and tissue-specific expression but was found consistently overexpressed in cancer samples compared to normal ones, and its expression is associated with increased invasion and metastasization in several tumor types. Indeed, high expression in primary tumor is a prognostic marker for metastasization and patient survival [81,82]. HOTAIR partakes to cell aggressiveness through several mechanisms that converge on EMT-related pathways. It was found overexpressed following TGFβ signal activation and it has been shown to have different roles in several tumors [83,84,85]. In malignant cervical cancer cells, HOTAIR over-expression led to upregulation of EMT-related genes, Notch1, Hes1 and p300. Thus, the authors sustained that HOTAIR may contribute to EMT-induced cancer aggressiveness via the activation of Wnt-Notch signalling [83]. In breast cancer cells, downregulation of miR-7, inhibitor of STAT3, may be attributed to HOTAIR transcriptional inhibition of HoxD10, a miR-7 expression promoter [84]. In ESCC, HOTAIR and WNT inhibitory factor (WIF1) expression were found inversely correlated. HOTAIR recruits PRC2 complex on WIF1 promoter to induce H3K27 methylation and inhibit transcription, indirectly activating the Wnt/βcatenin signaling pathway [85]. Noticeably, HOTAIR 5′ domain directly binds PRC2 and its 3′ domain binds lysine-specific demethylase 1 (LSD1), a component of the RE1-silencing transcription factor (CoREST/REST) repression complex, thus functioning as a scaffold and coordinator of two major histone-modifying complexes [28]. A recent work by Song et al. indeed demonstrated that at the E-CAD promoter HOTAIR switches H3K27 acetylation to methylation, blocking its expression and supporting EMT in gastric cancer (Figure 1A) [86].

The oncofetal **H19** is a lncRNA identified as overexpressed in a lot of cancer models compared to normal tissues, and to have multiple roles throughout tumorigenesis [87,88,89,90]. It responds concomitantly to several EMT-related signaling, among which TGFβ, HGF and Hypoxia/ Hypoxia Inducible transcription factor 1 (HIF1), which may in part explain why this lncRNA is particularly expressed in metastatic cells [89,90,91]. H19 forms a positive feedback loop with SLUG, promotes EZH2-mediated silencing of the E-CAD promoter, and functions as ceRNA for several anti-EMT miRNAs, further highlighting its relevance in EMT regulation [35]. Moreover, H19 sequence contains miR-675. Once processed, this miRNA support EMT enhancing cell invasion and proliferation impacting on several pathways among which the AKT/mTOR axis (Figure 1D) [92,93,94].

The list of the hypoxia-responding lncRNAs is increasing and the lncRNA regulator of reprogramming (**LncRNA-ROR**) is one of the most studied. It was originally identified in induced pluripotent stem cells (iPS) as a p53/stress response pathways inhibitor [95]. Its overexpression was then reported in several tumors. Consistently with its effect in iPS, its expression correlates with cell invasion capacity and expression of EMT markers [96,97,98]. As many other lncRNAs, its effect is attributed to its ceRNA role through which lncRNA-ROR sequesters and inhibits miRNAs targeting ZEB1/ZEB2 expression. Furthermore, in breast cancer cells this lncRNA sponges miR-145, which is a suppressor of the ADP-ribosylation factor 6 (ARF6), key regulator of the process of invasion [96].

Alternative splicing regulates the great majority of multi-exon genes in human. A work by Grelet et al. described the post-translational regulation of lncRNA expression through the control of the alternative splicing machinery [99]. Phosphatase 1 nuclear targeting subunit (PNUTS) gene can encode both for an mRNA and a lncRNA. Heterogeneous nuclear ribonucleoprotein E1 (hnRNP E1) impedes the alternative splicing that would lead to **lncRNA-PNUTS** production. Loss of this RNA-binding protein, induced by TGFβ, leads to an accumulation of the lncRNA form, which was found upregulated in tumor cells. lncRNA-PNUTS can interact with miR-205 and control the miR-205/ZEB/E-CAD axis, thus regulating EMT-mediated migration and invasion, besides tumor implantation, growth and metastasization in vivo [99].

**Linc00941** (also known as lncRNA-MUF) was recently described as EMT-promoter in hepatocellular carcinoma cells, where it exerts a double function, it sponges miR-34a, leading to SNAIL1 upregulation (Figure 1E) and binds Annexin2 (ANXA2) and Glycogen synthase kinase 3 beta (GSK3β). Its scaffold function influences β-catenin phosphorylation and nuclear location, promoting Wnt/β-catenin pathway (Figure 1B) [100]. Expression levels of LINC00941 were also correlated with invasion capacity, lymphatic metastasis, and stage of patients with gastric cancer [101].

## 6. Anti-EMT lncRNAs

As strongly suggested by the name, low expression in tumor (**LET**) is a lncRNA down-regulated in several cancer types. It was discovered as negatively regulated by hypoxia-induced histone deacetylase 3 (HDAC3) and its downregulation led to nuclear factor 90 (NF90) stabilization, which promotes cell invasion [102]. In nasopharyngeal carcinoma cells, in which LET inhibits cell proliferation and promotes apoptosis, its repression has been linked to EZH2-mediated H3K27 histone tri-methylation of LET promoter [103].

The lncRNA growth arrest-specific 5 (**GAS5**) has been reported to suppress tumor proliferation, migration, and the EMT in osteosarcoma directly sponging miR-221 to inhibit its function and enhance aplasia Ras homologue member I (ARHI) expression, which is a tumor suppressor gene in osteosarcoma, down regulated in several cancers [104]. A recent work by Liu et al. described GAS5 role also in pancreatic cancer, where it is significantly down-regulated compared to normal tissue and where this lncRNA reverses EMT and metastasization. Also, in this model, GAS5 functions as ceRNA for miR-221 affecting its targets, among which SOCS3, inhibitor of EMT and of the JAK2/STAT3 signaling [105]. GAS5-AS, the antisense RNA of GAS5, has been shown to be down regulated in NSCLC samples as compared to adjacent normal lung. Furthermore, their expression was shown to reversely correlate with TNM stages, tumor size, and lymph node metastasis suggesting a role of this noncoding transcript in this setting. Interestingly, these results indicated that both transcripts (GAS5 and GAS5-AS1) play tumor suppressive roles in NSCLC but influencing distinct pathways. GAS5 inhibits cell proliferation inducing apoptosis, GAS5-AS1 negatively controls cell migration and invasion, hypothesizing that the NSCLC cells with low expression of both lncRNAs may be even more aggressive than cells expressing low levels of one of them [106].

**Linc01186** was characterized as inhibitor of cell migration and invasion in a study performed to identify lncRNAs under TGFβ/SMAD3 regulation in lung cancer. Its transcription was indeed repressed by SMAD3, affecting the expression levels of several EMT markers [107]. It was recently described as tumor suppressor in thyroid papillary carcinoma (PTC) where it inhibited LATS1/YAP signaling pathway [108].

lncRNA-downregulated expression by hepatitis B virus X (**DREH**) was identified as down- regulated in HBx-related HCC compared to normal adjacent tissue. It acts as a tumor suppressor inhibiting cell proliferation in vitro and metastasization in vivo [109]. DREH was also found to interact and repress the intermediate filament Vimentin expression changing cytoskeleton architecture and inhibiting tumor metastasis [110].

Tumor suppressor candidate 7 (**TUSC7**) expression was found down regulated in HCC, NSCLC, pancreatic carcinoma, colorectal cancer, glioma and other tumor types [111,112,113]. In HCC, it counteracts EMT and invasion capacity sponging miR-10a, a well-known upregulated miRNA in this type of carcinoma, increasing the expression of the anti-EMT EPHA4 [111]. In other cell contexts, TUSC7 can function as ceRNA for other miRNAs which positively control EMT, like miR-211 and miR-371a-5p [113,114].

LEIGC lncRNA was described as downregulated in gastric cancer, where its overexpression can impair the invasiveness of cells. This transcript influences the expression of epithelial genes like E-CAD and mesenchymal makers but the mechanisms at the basis of its functioning are still unknown [115].

## 7. lncRNAs with Controversial Role in EMT

lncRNAs expression is restricted in time and space in a very stringent way. This high tissue-specificity grants a fine differential gene regulation and likely underpin a context specific differentiated function. Indeed, many lncRNAs have been described to have distinctive and often contradictory functions in different cellular contexts. This is true also for lncRNA that are engaged in EMT and cancer regulation.

One of the first lncRNA ever investigated is X-inactive specific transcript (**XIST**), found to be the master regulator of the X chromosome inactivation in mammals. Interestingly, an increasing number of studies pointed out that XIST expression is deregulated in multiple tumors. However, its role seems to be opposite in different types of tumors. A recent work by Chen et al. explored its role in metastasization of colorectal cancer (CRC). In this tumor cells, XIST was overexpressed and correlated with poor outcome. Knockdown experiments demonstrated that this lncRNA can modulate cell proliferation, invasion and EMT by sponging miR-200b-3p to modulate ZEB1 expression [116]. Another study in retinoblastoma (RB) showed that XIST supports proliferation, migration, invasion through the accomplishment of the EMT program and the blocking of apoptosis. It negatively regulates miR-101 to support ZEB1 and ZEB2 expression [117]. On the contrary, XIST was reported to be significantly down-regulated and its expression inversely correlated to advanced stage and lymph node metastasis in breast cancer [118]. Similar results were presented by Du et al. in prostate cancer, underlying that XIST role in carcinogenesis is largely tissue-dependent [119].

lncRNA Opa interacting protein 5-antisense RNA1 (**OIP5-AS1**) was reported to have controversial functions in different tumors. It is described to sponge miR-143-3p upregulating SMAD3 expression to contribute to cervical cancer cells metastasization, or as unfavorable predictor in hepatoblastoma in which it binds miR-186a-5p upregulating ZEB1 and promoting EMT, proliferation and metastasis [120,121]. On the contrary, other works indicate OIP5-AS1 as a protective factor, for instance in multiple myeloma cells where it binds the pro-EMT miR-410 preventing its inhibitory role on the oncosuppressor and SMAD repressor KLF10 [122].

Another example of contrasting functions of lncRNAs in EMT regulation is **SPRY4-IT1**. This is a long intergenic transcript which derives from an intron of Spry4, inhibitor of MAP-Kinase pathway. SPRY4-IT1 expression is induced in cancer compared to normal cells and this lncRNA has been shown to support EMT in osteosarcoma, gliomas, colorectal cancer, bladder cancer and others. On the contrary, its expression is down-regulated in NSCLC cells [123,124]. In this context, SPRY4 negatively regulates EMT with the cooperation of its associated lncRNA SPRY4-IT1. Also, in this case, EZH2 seems to be involved in the regulation of this lncRNA transcription [125].

ZNFX1 antisense RNA 1 (**ZFAS1**) lncRNA is aberrantly overexpressed and is reported as an oncogene in various tumors as melanoma, ESCC, NSCLC, gastric cancer, colon cancer. In gastric cancer its expression was associated with increased levels of ZEB1 and Wnt pathway, but other works have proposed opposing results in HCC [126,127]. A recent work showed that ZFAS1 inhibits miRNA-150 leading to activation of ZEB1, MMP14, and MMP16. By contrast, Wang et al. proposed the lncRNA as a oncosuppressor in HCC [128]. In breast cancer, ZFAS1 expression is impaired, and its activity linked to tumor suppressor function. Its overexpression diminished cell proliferation arresting cell cycle and inducing apoptosis and inhibited cell migration and invasion by regulating EMT [129].

lncRNA in nonhomologous end joining pathway 1 (**LINP1**) was reported to be transcriptionally inhibited by TGF-β1 in a SMAD4-dependent manner in lung cancer cells. LINP1 suppressed EMT controlling cancer cell migration and invasion [130]. On the contrary, in breast cancer, LINP1 was reported to be regulated by p53 and EGFR signaling and its silencing increased tumor-cell response to radiotherapy [131].

Maternally expressed gene 3 (**MEG3**) is a lncRNA object of numerous recent works reporting both pro- and anti-cancer activity of this transcript, depending on the context. In lung cancer, MEG3 has been proposed as a positive regulator of EMT. Mechanistically, this lncRNA regulates recruitment of EZH2 and JARID2, another component of the PRC2 complex, on E-CAD and miR-200 family loci to impair their transcription [132]. By contrast, a study in gastric cancer showed that MEG3 expression is down-regulated in tumor compared to normal tissue and that, in vitro, MEG3 suppressed cell motility, negatively regulating miR-21, a pro-EMT miRNA [133]. According to this work, others reported an anti-EMT role of this lncRNA in additional tumor models but describing different mechanisms of action [134,135].

BRAF-activated non-protein coding RNA (**BANCR**) was first described as overexpressed in melanoma cells where it is required for melanoma cell migration [136]. In colorectal cancer, BANCR induced EMT through the MEK/ERK signaling, promoting cancer cells aggressiveness. A similar function was recently reported in PTC [137,138]. BANCR positive regulation of EMT and invasion capability was also reported in breast cancer cells [139]. On the contrary, in NSCLC its expression was significantly lower in advanced tumor stages and in metastatic tumors, and its down regulation correlated with a shorter overall survival in patients. BANCR overexpression led to inhibition of cell migration and invasion and promotion of apoptosis in vitro, and in a significant reduction in the number of lung metastasis in vivo. BANCR inhibitory effects on cell migration and invasion were associated with EMT suppression, thus indicating this lncRNA as a positive prognostic factor [140].

## 8. lncRNAs Diagnostic, Prognostic and Therapeutic Perspectives

### 8.1. lncRNAs as Biomarkers

lncRNAs have been proven to be a useful tool to understand the molecular mechanisms at the basis of physiological and pathological processes. A current challenge is to correctly contextualize their expression and activity in order to exploit their value as biomarkers. lncRNAs expression is stringently regulated and selectively activated in a time and space dependent manner. Furthermore, differently from what previously thought, lncRNAs are characterized by consistent stability [142,143]. These characteristics and the presence of this transcripts in tissues and circulating fluids opened a new field of study aiming to evaluate their putative role in this sense.

Metastatic spreading is the major event determining cancer-dependent death and EMT is an early key pathway in this process. Thus, growing efforts are made to understand if lncRNAs could be used to predict tumor metastatic potential. Most of lncRNAs discussed above were described deregulated in tumor samples compared to normal tissues and some of them have shown significant correlation with tumor metastasis and reduced patients’ overall or disease-free survival.

For instance, PVT1 was proposed as diagnostic and prognostic marker in primary acute promyelocytic leukemia (APL) [144]. A recent work proposed the combination of the oncogenic MYC, PVT1, and another lncRNA transcribed from the same genomic locus as prognostic marker also in t(8;21) associated acute myeloid leukemia (AML). They found the signature linked to all characteristics of the progression of the disease. Moreover, high expression of the three transcripts were associated to high minimal residual disease, suggesting PVT1 involvement in multidrug resistance [145].

The serum levels of lncRNA H19, together with miR-204, miR-182 were recently proposed as prospective plasma biomarkers to detect GC and its progression from ulcer caused by *H. pylori*. The authors indeed showed that serum lncRNA H19 has a high diagnostic accuracy, specificity and sensitivity [146]. Another work, on bladder cancer (BC), showed a positive correlation among serum exosomal H19 with total H19 level in paired cancer tissues. Exosomal H19 levels were significantly higher in serum of BC patients compared to control samples and higher H19 expression correlated with poor overall survival. Detection of serum exosomal H19 was thus proposed as non-invasive diagnostic and prognostic biomarker for these patients [147]. The detection of H19 to aid patients’ stratification is proposed in several other tumor settings [148,149].

Deep sequencing analysis of patients’ DNA samples discovered an unexpected large amount of mutations and copy number variations mapping within noncoding genomic regions. The meaning of many of these alterations remain difficult to understand and a main future challenge will be to discriminate, among all noncoding alterations, those that have biological relevance and a real impact on cancer behavior [150,151].

To date, an increasing number of meta-analyses are trying to put together a large gene expression dataset to define if and which lncRNAs have a prognostic value in different tumor types, with promising results [152,153,154,155]. In one of these studies, the authors performed an in-depth transcriptomic analysis using TCGA breast cancer RNA-seq data. They found 215 lncRNAs whose transcription is dysregulated in this type of cancer and independently validated a discovery signature. They also reported an association among lncRNAs expression, some clinical features and tumor relapse. lncRNAs part of the signature were mainly associated to key molecular pathways like EGFR, PI3K, MAPK, and E2F1. The lncRNA profiling could also distinguish between ER+ and ER− tumors allowing stratification [152].

A similar analysis of lncRNA expression profiles was performed on Diffuse large B-cell lymphoma (DLBCL) cohort from Gene Expression Omnibus (GEO) datasets. The discovery cohort led to a set of 17 of the 156 differentially expressed lncRNAs that were chosen as putative biomarkers able to discriminate among major tumor subtypes. Patients expressing the 17 lncRNAs signature presented significantly worse clinical outcome. The lncRNA composing the signature were mainly involved in immune-associated processes and chemokine signaling pathway. Interestingly, this signature was proposed both for DLBCL classification and prognosis [154].

Another branch of study in this field is focusing on evaluating the clinical potential of circulating lncRNAs as cancer biomarkers, but to date a global consensus on procedures and detection protocols lacks and needs to be fully elucidated before giving potential answers [156]. It is now clear that single tumor-associated circulating lncRNA detection is not a powerful prognostic nor diagnostic tool. On the contrary, signatures composed by a combination of various circulating lncRNAs can considerably promote the efficiency of some cancer detection. In a work performed in gastric cancer, the authors proposed a signature composed of three circulating lncRNAs which could distinguish cancer patients from healthy controls, with great sensitivity and specificity, suggesting a putative use of the signature for diagnosis [157].

Tong et al. found that the plasma levels of three lncRNAs of the 10 evaluated could discriminate healthy from ESCC patients. One of these lncRNAs improved the diagnostic specificity of the Squamous Cell Carcinoma Antigen (SCCA), a diagnostic marker currently in use in this setting. Interestingly, this combination effectively detects more than 80% of ESCC at an early stage [158].

Several works demonstrated the feasibility of using circulating lncRNAs as cancer biomarkers but many preanalytical and analytical factors are slowing the translation into clinic. All the steps of the process must be set and standardized. Thus, great efforts should be made to improve analytical approaches, which should be tailored on the peculiarity of these molecules.

### 8.2. lncRNAs as Therapeutic Targets

The emerging role of lncRNAs as regulators of both oncosuppressive and oncogenic pathways, including EMT, raises the challenge of efficiently targeting them both to explore their actual function in cancer biology and to set-up possible strategies to counteract their effects in cancer patients. Currently, a variety of approaches for lncRNAs targeting are under investigation, even if major limitations in this context remain [159].

- *RNA interference (RNAi)*. It allows specific knockdown of target RNAs introducing exogenous complementary RNAs and can be achieved through different agents like siRNA (small interfering RNAs), shRNAs (short hairpin RNAs), and miRNAs. The interference is accomplished through the activation of RNAase III enzyme Dicer, a RNA-induced silencing complex (RISC) and Argonaute (AGO2) endonuclease [160]. Several pharmaceutical companies tried to ameliorate siRNA-based drugs working on different aspects, among which chemical modification to counteract their degradation. However, in vivo experiments using RNAi did not lead to striking results. This was principally due to lack of efficient delivery methods, like liposomes, nanoparticles or viruses for proper cellular uptake and to protect RNAs from degradation. Further improvements are surely needed for safe and effective use of RNAi-based therapies, but some of them already reached clinical trials [161,162].
- *Antisense oligonucleotides (ASOs)*. 13–25 nt DNA sequences designed to bind complementary RNA targets to cleave them through the activation of RNase H or to promote splicing alterations [163]. The advantage of these molecules is that, exploiting RNase H mechanism, can easily target nuclei-enriched RNAs, unlike the above described RNAi. ASOs were chemically modified to avoid degradation and used in clinical trials to target mRNAs. Few of them are approved by the Food and Drug Administration (FDA) for hypercholesterolemia, so they are emerging also as a tool for lncRNAs targeting [164]. Unfortunately, modified ASOs-mediated RNase H induction is scarce, thus to achieve better results, a series of subsequent modifications were performed. This led to the development of chimeric RNA-DNA-RNA ASOs referred to as gapmers, locked nucleic acids (LNAs) and D-constrained ethyl (cRt) which entered cancer-related clinical trials [165,166,167,168]. AntagoNATs, particular type of ASOs, are designed to target Natural Antisense RNAs (NATs), a subset of ncRNAs overlapping protein-coding genes, and transcribed in the antisense direction [169,170]. NATs often negatively impact on protein-coding genes expression, acting in cis. Thus, targeting these molecules could upregulate nearby genes expression, being a valuable resource to re-express oncosuppressor genes [171].
- *Morpholinos*. 25-nt DNA analogs initially used for loss of function studies in zebrafish, have then been used to regulate miRNAs activity, to prevent mRNAs translation or to promote pre-mRNAs alternative splicing [172,173]. Even though preclinical studies using morpholisnos are limited, some of them are clinically approved for Duchenne dystrophy thus they could be a useful tool to counteract lncRNAs [174].
- *Aptamers*. Single-stranded nucleic acids engineered through repeated rounds of in vitro selection called systematic evolution of ligands by exponential enrichment (SELEX) to bind small molecules, proteins, nucleic acids, but also cells and tissues [175]. Aptamers were developed as an alternative to antibodies to target every molecular target without elicit immunological reactions in therapeutic, imaging and diagnostic applications [176,177,178,179]. The first RNA aptamer was approved by FDA in 2004 to treat macular degeneration [180]. To date, a number of aptamer-based drugs have been evaluated in cancer clinical trials [181]. Cancer-targeting aptamers have been used for the selective recognition and elimination of tumorigenic cells and for new biomarkers discovery. Moreover, aptamers were used to block or activate immune receptors and cytokines, modulating intrinsic immune responses against cancers, thus some works are proposing these molecules as a promising support for immunotherapies [182,183,184].
- *Clustered Regularly Interspaced Short Palindromic Repeats (CRISPR) effectors*. With recent advances in CRISPR/Cas9 genome editing methods, lncRNAs transcriptional silencing became a realistic perspective. CRISPR interference (CRISPRi) allows the exploitation of dead-Cas9, a mutated enzyme lacking endonuclease activity unable to provoke double-strand brakes on DNA, fused to a transcriptional repressor. This fused complex is targeted to the genomic region of interest with guide RNAs. A recent work by Liu et al. used this technique to specifically inactivate a series of lncRNAs in different cell lines, underlying the differential essentiality of these transcripts in different settings [185]. Another method recently proposed implicates the use of CRISPR/Cas13. Cas13 differs from Cas9 because binds and cleaves RNA instead of DNA, which could be a promising tool for RNA-directed therapies [186]. Several preclinical studies used CRISPR effectors to edit DNA or RNA but efficient translation into clinical practice remains to be verified [187,188].
- *Small molecules*. Low molecular weight organic compounds able to enter the plasma membrane and interact with their targets are widely used in clinic. Interestingly, such molecules could be developed to target secondary or tertiary RNA structures, but this field is still on its infancy [189,190].

Some works described the use of EMT-related lncRNA-targeting strategies in preclinical subsets. For instance, subcutaneous injection of MALAT1-targeting ASOs in a mouse model of metastatic luminal B breast cancer resulted in the formation of cystic and non-metastatic tumors. Since MALAT1 is highly expressed in metastases of human breast cancer, the authors suggested further clinical investigation to evaluate this therapy to support breast cancer treatment [191].

However, despite the great enthusiasm for nucleic-acids based therapies and some therapeutic success, such strategies were developed over 40 years ago and pitfalls still exist. Plasma membrane crossing and cellular nucleases recognizing non-self RNAs, such as Toll-like receptors and RNA helicases, are still major caveats, limiting the efficacy of this methods. Moreover, the problem of off-targets is not negligible and still raises perplexities.

## 9. Conclusions

LncRNAs are emerging as key players in all cellular contexts and their importance in cancer initiation and progression is relevant, but the study of their mechanisms of action and the evaluation of which transcripts are valuable target remain to be addressed. Furthermore, the development of techniques both to study lncRNAs functionally and to set targeting strategies, overcoming off-target and toxicity effects, is needed to translate lncRNAs in a clinical setting.

## Figures and Tables

**Table 1:** Summary of the described lncRNAs involved in EMT regulation.

lncRNA	Role in EMT	Pathways Involved	Molecular Mechanism	Refs
**BANCR**	pro-EMT	MAPK; NFκB	Unknown. E-CAD downregulation; Vimentin, N-CAD, p-ERK/MEK and p-ERK1 upregulation	[136,137,138,139]
**BANCR**	anti-EMT	MAPK; NFκB	Unknown. E-CAD upregulation; Vimentin and N-CAD downregulation	[140]
**DREH**	anti-EMT	-	Binds Vimentin changing cytoskeleton architecture	[109,110]
**GAS5**	anti-EMT	JACK/STAT3	ceRNA for miR-221;ARHI upregulation; SOCS3 upregulation	[104,105]
**GAS5-AS1**	anti-EMT	-	Unknown. Its overexpression reduces N-CAD, Vimentin and ZEB1 expression.	[106]
**H19**	pro-EMT	TGF-β; Wnt/β-catenin; HIF1α; AKT/mTOR	ceRNA for several anti-EMT miRNAs; contains miR-675; EZH2 mediated silencing of E-CAD	[87,88,89,90,91,92,93,94]
**HOTAIR**	pro-EMT	TGF-β; Wnt/β-catenin; JAG/Notch	ceRNA for miR-7; STAT3 upregulation; coordinator of PRC2 and LSD1 on E-CAD promoter; indirect upregulation of Wnt targets	[81,82,83,84,85,86]
**HOTTIP**	pro-EMT	Wnt/β-catenin; TGF-β	ceRNA for miR-30b; upregulation of SNAIL; transcriptional regulation of HOXA genes	[54,55,56]
**HOXA11-AS**	pro-EMT	Wnt/β-catenin; PI3K/AKT; TGF-β	ceRNA for miR-214-3p, miR-200b, miR-140-5p; transcriptional regulation of HOXA genes	[33,51,52,53]
**LEIGC**	anti-EMT	-	Unknown. Influences EMT markers expression	[115]
**LET**	anti-EMT	HIF1α	Increases NF90 degradation by the proteasome	[102,103]
**Linc00941**	pro-EMT	TGF-β; Wnt/β-catenin	ceRNA for miR-34a; SNAIL1 upregulation; scaffolf for ANXA2 and GSK3β to promote Wnt pathway	[100,101]
**Linc01186**	anti-EMT	TGFβ; Hippo	Unknown. Inhibits YAP1 while increasing LATS1 expression	[107,108]
**LINP1**	pro-EMT	EGFR; p53	Scaffold for KU80 and DNA-PKcs coordinating the NHEJ and supporting cell growth and EMT	[131]
**LINP1**	anti-EMT	TGFβ	Unknown. Its upregulation blocks E-CAD downregulation and SNAIL and Vimentin over-expression	[130]
**lncRNA-ATB**	pro-EMT	TGFβ; IL-11/STAT3	ceRNA for miR-200 and miR-141-3p; upregulation of ZEB1-2; stabilization of STAT3	[41,42,43,44,45,46]
**lncRNA-HIT**	pro-EMT	TGFβ; Rb/E2F	ceRNA for miR-200; upregulation of ZEB1-2; cell cycle regulation through E2F1	[47,48,49,50]
**lncRNA-PNUTS**	pro-EMT	TGF-β	ceRNA for miR-205; upregulation of ZEB1 and ZEB2	[99]
**lncRNA-ROR**	pro-EMT	HIF1α; p53	ceRNA for miR-145, miR205; upregulation of ZEB1, ZEB2 and ARF6	[95,96,97,98]
**lnc-SNHG1**	pro-EMT	TGFβ; Insulin/IGF1R; Wnt/β-catenin	ceRNA for miR-497, miR-302/372/373/520	[39,40]
**MALAT1**	pro-EMT	TGF-β; Wnt/β-catenin; PI3K/AKT; MAPK	ceRNA for miR-205, miR-204, miR-1, miR-30a; upregulation of SLUG and STAT3; regulation of speckles; EZH2-mediated regulation of E-CAD transcription	[29,61,62,63,64,65,66,67,68,69,70,71,72,73,74,75,76]
**MEG3**	pro-EMT	TGFβ	PRC2 mediated E-CAD and miR-200 family transcription impairment	[132]
**MEG3**	anti-EMT	TGFβ	ceRNA for miR-21; SPHK1 downregulation	[133,134]
**NEAT1**	pro-EMT	STAT3; PI3K/AKT	ceRNA for miR-204 and miR-101; regulation of paraspeckles; transcriptional regulation of GATA3	[57,58,59,60,61,62]
**OIP5-AS1**	pro-EMT	TGFβ	ceRNA for miR-143-3p and miR-186a-5p; SMAD3 and ZEB1 upregulation	[120,121]
**OIP5-AS1**	anti-EMT	PI3K/AKT	ceRNA for miR-410; KLF10 upregulation	[122]
**PVT1**	pro-EMT	TGFβ; p21/p53	phosphorylation of SMAD2/3; downregulation of p21	[37,38]
**SPRY4-IT1**	pro-EMT	TGFβ	SNAIL stabilization	[123,124,141]
**SPRY4-IT1**	anti-EMT	TGFβ	Downregulation of E-CAD and Vimentin	[125]
**TUG1**	pro-EMT	TGF-β	ceRNA for miR-145 and miR-300	[79]
**TUSC7**	anti-EMT	Integrinβ; p53	ceRNA for miR-10a, miR-211 and miR-371a-5p; downregulation of several EMT markers	[113,114]
**UCA1**	pro-EMT	TGF-β; Wnt/β-catenin; MAPK; AKT/mTOR; JAG/Notch	ceRNA for miR-582-5p and others; upregulation of ZEB1 and ZEB2; autophagy impairment	[77,78]
**XIST**	pro-EMT	TGFβ	ceRNA for miR-200b-3p and miR-101; ZEB1 and ZEB2 upregulation	[116,117]
**XIST**	anti-EMT	MAPK; Wnt/β-catenin	miR-155 and miR-23a; RKIP	[118,119]
**ZEB1-AS1**	pro-EMT	TGF-β	ceRNA for miR-149-3p, miR-200; MLL1-mediated ZEB1 transcriptional regulation; E-CAD transcriptional downregulation; N-CAD, MMP2, MMP9 upregulation	[33,71,72,73,74]
**ZEB2-AS1**	pro-EMT	TGF-β	prevents splicing of ZEB2 mRNA enhancing its translation	[75,76]
**ZFAS1**	pro-EMT	Wnt/β-catenin	ceRNA for miRNA-150; ZEB1, MMP14 and MMP16 upregulation; Wnt pathway activation	[126,127]
**ZFAS1**	anti-EMT	-	miR-9 transcriptional regulation	[128]
